# Cost‐Effectiveness of Sequential Teriparatide/Alendronate Versus Alendronate‐Alone Strategies in High‐Risk Osteoporotic Women in the US: Analyzing the Impact of Generic/Biosimilar Teriparatide

**DOI:** 10.1002/jbm4.10233

**Published:** 2019-11-13

**Authors:** Takahiro Mori, Carolyn J Crandall, David A Ganz

**Affiliations:** ^1^ Department of Health Services Research, Faculty of Medicine University of Tsukuba Tsukuba Ibaraki Japan; ^2^ Health Services Research and Development Center University of Tsukuba Tsukuba Ibaraki Japan; ^3^ Department of General Internal Medicine Eastern Chiba Medical Center Togane Chiba Japan; ^4^ Division of General Internal Medicine and Health Services Research, Department of Medicine, David Geffen School of Medicine University of California, Los Angeles Los Angeles CA USA; ^5^ Geriatric Research, Education and Clinical Center and HSR&D Center for the Study of Healthcare Innovation, Implementation and Policy, Veterans Affairs Greater Los Angeles Healthcare System Los Angeles CA USA; ^6^ Division of Geriatrics, Department of Medicine, David Geffen School of Medicine University of California, Los Angeles Los Angeles CA USA; ^7^ Health Unit RAND Corporation Santa Monica CA USA

**Keywords:** ALENDRONATE, BIOSIMILAR, COST‐EFFECTIVENESS ANALYSIS, GENERIC, OSTEOPOROSIS, TERIPARATIDE

## Abstract

Teriparatide, currently only available in brand form in the United States, is a costly drug approved for the treatment of postmenopausal osteoporotic women who are at high risk of fracture. Because market exclusivity for brand teriparatide expired in August 2019 in the US, we sought to understand the potential health economic impact of the availability of generic or biosimilar (generic/biosimilar) teriparatide. We examined the cost‐effectiveness of daily teriparatide for 2 years followed by weekly alendronate for 10 years (ie, sequential teriparatide/alendronate) compared with alendronate alone for 10 years in community‐dwelling white osteoporotic women with prior vertebral fracture at ages 65, 70, 75, and 80. Using an updated version of previously validated Markov microsimulation models, we obtained incremental cost‐effectiveness ratios (ICERs) (dollars [$] per quality‐adjusted life year [QALY]) with a willingness‐to‐pay (WTP) of $150,000 per QALY from a societal perspective with a lifelong time horizon. In the base case, we estimated the annual cost of teriparatide to be $20,161, based on the assumption of 10% brand usage (at a cost of $27,618) and 90% generic/biosimilar usage (priced 30% lower than brand). The ICERs of sequential teriparatide/alendronate compared with alendronate alone were greater than $280,000 per QALY at all ages examined. In deterministic sensitivity analyses, results were sensitive to teriparatide's cost, with the cost of a generic/biosimilar product needing to be 65% to 85% lower than brand for sequential teriparatide/alendronate to be cost‐effective. In probabilistic sensitivity analyses, under the assumption that the annual cost of teriparatide was $20,161, the probabilities of sequential teriparatide/alendronate being cost‐effective were less than 4% at a WTP of $150,000 per QALY. In conclusion, among community‐dwelling older osteoporotic women with prior vertebral fracture in the US, even with the potential availability of generic/biosimilar teriparatide, sequential teriparatide/alendronate would not be cost‐effective unless the cost of generic/biosimilar teriparatide were heavily discounted with respect to the current brand cost. © 2019 The Authors. *JBMR Plus* published by Wiley Periodicals, Inc. on behalf of American Society for Bone and Mineral Research.

## Introduction

Teriparatide is a recombinant form of parathyroid hormone that stimulates bone formation and activates bone remodeling. It has been approved by the US Food and Drug Administration (FDA) since 2002 for the treatment of postmenopausal women with osteoporosis who are at high risk of fracture, defined as a history of osteoporotic fracture or multiple risk factors for fracture.^1^ Teriparatide is the most expensive drug for osteoporosis treatment in the United States, with a wholesale acquisition cost (WAC) for the daily form of $3426.50 for a 28‐day supply as of June 2019.^2^ Although only the brand version of teriparatide was available in the US at the time of this writing, the patent for teriparatide use did expire in August 2019.^3^

A less‐expensive version (ie, generic or biosimilar) of teriparatide is expected to arrive in the US market in the future. Generic drugs have the same active ingredients as the brand drug for achieving the identical condition (ie, same requirements for identity, strength, purity, and quality).^4^ In contrast, biosimilar drugs are biological products that are highly similar to FDA‐approved biologic reference products and have no clinically meaningful differences from the reference products.^5^ Biosimilars of teriparatide have been approved in other countries.^3^ Throughout this article, we use the term “generic/biosimilar” to refer to either a generic or biosimilar product that is less expensive than the brand product, with the efficacy remaining the same.

A 2006 US cost‐effectiveness analysis compared daily s.c. teriparatide for 2 years followed by daily oral alendronate for 5 years (ie, sequential teriparatide/alendronate) with alendronate alone for 5 years in women with severe osteoporosis.^6^ The authors concluded that sequential teriparatide/alendronate was not cost‐effective, mainly because teriparatide was expensive.

Assuming that teriparatide becomes less expensive after generic/biosimilar versions become available on the market, we sought to understand the potential economic impact of the availability of generic/biosimilar teriparatide in the US.

## Methods

The reporting of this economic evaluation followed the Consolidated Health Economic Evaluation Reporting Standards (CHEERS), the recommendations of the Second Panel on Cost‐Effectiveness in Health and Medicine, and the recent recommendations for the conduct of economic evaluation in osteoporosis (Supplemental Table 1).^7^, ^8^, ^9^ We used an updated version of previously validated Markov microsimulation models^10^, ^11^ to perform a cost‐effectiveness analysis among hypothetical cohorts of community‐dwelling osteoporotic women with previous vertebral fracture in the US at various ages of therapy initiation (65, 70, 75, and 80 years old). We chose to focus on white women for this analysis because they represent a high‐risk group for fracture.^10^ We estimated total costs in 2018 US dollars and quality‐adjusted life years (QALYs) to obtain incremental cost‐effectiveness ratios (ICERs), which represent the cost per QALY gained for one strategy compared with the other, over a lifetime horizon (until a participant reached age 105 or died). We evaluated cost‐effectiveness from two perspectives (societal as a primary analysis and health care sector) and provided an impact inventory (Table 1).^8^ We set a willingness‐to‐pay (WTP) of $50,000, $100,000, or $150,000 per QALY.^12^, ^13^ All costs and health benefits were discounted at 3% per year for the base case.^8^ We used Treeage Pro Suite 2018 (Treeage Software Inc., Williamstown, MA, USA) to program the model.

**Table 1:** Impact Inventory

Type of impact	Perspective	
	Societal	Health care sector
**Formal health care sector**		
**Health outcomes (effects)**		
Longevity effects	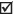	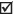
Health‐related quality of life effects	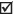	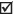
Other health effects (eg, adverse events)	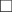	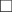
**Medical costs**		
Medications	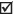	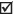
Physician visits	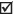	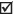
Future related costs^a^	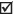	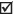
Future unrelated medical costs	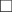	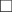
**Informal health care sector**		
Patient‐time costs	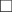	Not applicable
**Non‐health care sector**		
Cost of long‐term care after hip fracture	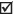	Not applicable
Cost of unpaid lost productivity caused by hip fracture	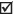	Not applicable

a Both payer and patients.

### Markov model structure

Each cycle lasts for 1 year, and every participant may sustain a hip, clinical vertebral, wrist, or other osteoporotic fracture (ie, humerus, distal forearm other than wrist, pelvis, tibia/fibula, or femur other than hip) during each cycle. Each individual can have only one fracture per cycle, and can have a maximum of two hip fractures, and an unlimited number of clinical vertebral, wrist, or other osteoporotic fractures over the entire time horizon. We used tracker variables for fractures and interventions to incorporate memory of previous events from one cycle to the next in the model. The details of the model structure can be found in our previously published work and its technical appendix.^10^

Model inputs included total costs, health‐related quality of life, and transition probabilities between the four Markov states (Table 2). Literature searches were performed extensively for all the parameters in the model, and inputs were derived from published sources (ie, meta‐analyses, systematic reviews, observational studies, cost‐effectiveness analyses, websites, or a book) that were considered as most relevant, high‐quality, and up‐to‐date. Our own assumptions were chosen only if no reliable published estimate was available.

**Table 2:** Key Model Parameters

	Value	Range	Distribution	Ref
**Teriparatide**				
**Efficacy**				
Hip fracture	0.42	0.10–1.0	Triangular	18
Clinical vertebral fracture	0.30	0.16–0.55	Beta	18
Wrist fracture	0.24	0.02–1.0	Triangular	19
Other osteoporotic fracture	0.50	0.32–0.78	Beta	18
**Persistence and adherence (%)**				
Persistence, 12 months	63.4	±50%^a^	Triangular	23
Persistence, 24 months	40.8	±50%^a^	Triangular	23
Adherence, 12 months	54.4	±50%^a^	Triangular	23
Adherence, 24 months	39.8	±50%^a^	Triangular	23
**Alendronate**				
**Efficacy**				
Hip fracture	0.45	0.27–0.68	Beta	18
Clinical vertebral fracture	0.50	0.33–0.79	Beta	18
Wrist fracture	0.82	0.25–1.0	Triangular	19
Other osteoporotic fracture	0.78	0.66–0.92	Beta	18
**Persistence and adherence (%)**				
Persistence, 12 months	38.7	±50%^a^	Triangular	23
Persistence, 24 months	23.7	±50%^a^	Triangular	23
Adherence, 12 months	31.3	±50%^a^	Triangular	23
Adherence, 24 months	22.8	±50%^a^	Triangular	23
**Costs (2018 US dollars)**				
**Formal health care sector**				
Alendronate, annual	205	86–324	Triangular	2
Teriparatide, annual	20,161	4005–22,646^b^	Triangular	2
Physician visit (CPT code 99213)	74	NA	NA	33
DXA scan (CPT code 77080)	100	49–150	Triangular	33
**Treatment costs**				
Hip fracture	29,986	25,677–42,913	Log‐normal	10
Clinical vertebral fracture	8325	5775–15,975	Log‐normal	10
Wrist fracture	4577	2543–10,674	Log‐normal	10
Other osteoporotic fracture	14,144	10,086–26,314	Log‐normal	10
**Non‐health care sector**				
Annual long‐term care after hip fracture	2577	0–5154	Triangular	10
Unpaid lost productivity caused by hip fracture, age 65–69	1690	±50%^a^	Triangular	36
Unpaid lost productivity caused by hip fracture, age 70–74	1005	±50%^a^	Triangular	36
Unpaid lost productivity caused by hip fracture, age 75–80	357	±50%^a^	Triangular	36
**Relative risks of subsequent fractures associated with prior vertebral fracture**				
Hip fracture	2.3	2.0–2.8	Gamma	39
Clinical vertebral fracture	4.4	3.6–5.4	Gamma	39
Wrist fracture	1.4	1.2–2.7	Gamma	39
Other osteoporotic fracture	1.8	1.7–1.9	Gamma	39

The other model parameters can be found in our earlier publication and its technical appendix.^10^ CPT = Current Procedural Terminology.

a Sensitivity values 50% lower and 50% higher in relative terms than the base case value, which was based on our own assumptions.

b $4005 was based on the assumption that 90% took generic/biosimilar product and the price discount for generic/biosimilar product was 95% relative to the brand product. $22,646 was based on the assumption that 60% took a generic/biosimilar product and the price discount for a generic/biosimilar product was 30% relative to the brand product.

### Efficacy of treatments

We compared the cost‐effectiveness of sequential teriparatide/alendronate, which in this study was defined as daily s.c. teriparatide for 2 years followed by weekly oral alendronate for 10 years, with alendronate alone for 10 years. Teriparatide is approved for up to 2 years of use because of the lack of proven efficacy after 2 years of use and a potential risk of osteosarcoma based on observations in rats. Bisphosphonates are typically prescribed after the completion of teriparatide to prevent bone density decline and loss of fracture efficacy.^1^, ^6^, ^13^, ^14^ Although the optimal duration of alendronate has not been determined, those who are at high risk for osteoporotic fractures may benefit from 10 years of therapy.^15^, ^16^, ^17^ In a sensitivity analysis, we examined the use of teriparatide for 2 years followed by alendronate for 5 years compared with alendronate alone for 5 years.

Data from systematic reviews and network meta‐analyses were used to obtain the efficacy of teriparatide and alendronate compared with placebo in reducing the risks of fragility fractures for those with osteoporosis.^18^, ^19^, ^20^ We primarily used Murad and colleagues' study^18^ based on the better AMSTAR (A Measurement Tool to Assess Systematic Reviews) score (10 out of 11) than the other studies in which AMSTAR scores ranged from 3 to 7 out of 11, reported by a systematic review.^20^ Although in Murad and colleagues' study, teriparatide had a 95% Bayesian credible interval of pair‐wise OR for hip fracture that crossed 1.0, in the base case we assumed teriparatide reduced the risk of hip fracture, as the study reported that the probability of teriparatide being ranked as the most efficacious in reducing the risk of hip fracture was the highest (ie, 42%) of all the other treatments including denosumab, oral or i.v. bisphosphonates. As Murad and colleagues' study did not present the pair‐wise OR for wrist fracture, we referred to Freemantle and colleagues' study,^19^ which has the second‐best AMSTAR score (7 out of 11). Although the 95% CIs of relative risks of teriparatide or alendronate for wrist fracture also crossed 1.0, we also assumed these treatments were efficacious in reducing the risk of wrist fracture in the base case, as the probabilities of relative risk less than 1.0 were estimated to be 91% for teriparatide and 67% for alendronate. In sensitivity analyses, we set the upper limits of intervals to 1.0 for those that crossed 1.0 (ie, teriparatide for hip or wrist fractures, or alendronate for wrist fracture).

Persistence and adherence are important factors that influence the outcome of the treatment of osteoporosis,^21^ where persistence refers to “the duration of time from initiation to discontinuation of the therapy” and adherence refers to “the extent to which a patient acts in accordance with the prescribed interval and dose of a dosing regimen.”^22^ We estimated persistence and adherence rates of alendronate and teriparatide based on Durden and colleagues' recent observational study in the US setting, in which persistence and adherence rates for 12 months and 24 months of teriparatide were higher than those of weekly oral alendronate.^23^ These results were consistent with a previous study in Germany, in which 12‐month persistence and adherence rates were higher with daily teriparatide than with weekly alendronate.^24^ As Durden and colleagues' study in the US setting reported information on persistence and adherence rates at 12 months and 24 months, we assumed a linear decline of persistence and adherence rates beyond 2 years up to 5 years. We assumed that those who took alendronate for 5 years continued to take alendronate for 10 years with the same adherence rate as the 5th year from the sixth year onward.

Adherence rates of weekly oral alendronate were higher in clinical trials (mostly greater than 80%, as high as 100%) than observational studies that reflected actual clinical settings.^21^ We estimated the relative efficacy of alendronate in the community by assuming a linear relationship between relative risk reduction and adherence. For example, if we assumed the adherence rate was 60% in the community, the relative efficacy in the community was estimated to be 0.75, based on 60% (community adherence)/80% (adherence of clinical trials).^10^, ^11^ We applied the same assumption to teriparatide for consistency.

We assumed that alendronate was efficacious at reducing the risk of fractures from the first year through 10th year, and that the risk of fractures after completing the therapy returned to rates in the absence of alendronate over 10 years in a gradual linear fashion (ie, offset effects).^10^, ^11^ Similarly, we assumed that teriparatide had efficacy from the first year through the second year and the risk for fractures returned to rates in the absence of teriparatide after 3 years, consistent with a recent cost‐effectiveness analysis regarding teriparatide.^14^ To keep the model parsimonious, we assumed that each individual obtained benefits of fracture prevention if she persisted in taking the treatment to the end of each cycle (ie, 1 year). In addition, for those who discontinued either alendronate or teriparatide before the predetermined period (ie, 2 years for teriparatide, 10 years for alendronate), the offset effects after discontinuation of therapy were assumed to be proportional to the length of the treatment periods.

Furthermore, we assumed that those who were on treatments and developed a fracture continued a total course of treatment with the same persistence and adherence, as there does not appear to be an association between prior history of fracture and persistence or adherence with osteoporosis therapy.^21^ Those who initially did not start treatments continued not to be on treatments after a fracture in this study to keep the model parsimonious.^10^, ^11^

### Costs

We divided costs into formal health care sector, informal health care sector, and non‐health care sector costs (Table 1). We assumed that costs were identical regardless of age, unless specified otherwise. All costs were presented in 2018 US dollars unless specified otherwise, using the Consumer Price Index for Medical Care for All Urban Consumers or the Consumer Price Index for All Items for All Urban Consumers, depending on the nature of the costs.^25^

#### *Formal health care sector*

We included the costs (the sums of payments by third‐party payers and by patients out‐of‐pocket) of alendronate, teriparatide, physician visits, DXA scans, and fracture‐related treatments.

It is challenging to estimate the costs of medications in the US health‐care system, as there is no generally applicable schedule of reimbursements available for medications. To estimate the cost of alendronate, we used 64% of the average wholesale price (AWP) of a brand product as the cost of a brand medication and 27% of the AWP of a generic product as the cost of a generic medication, as recommended by the U.S. Department of Veterans Affairs Health Economics Resource Center (VA HERC).^26^ The annual cost of generic alendronate was then estimated to be $86 ($6.13833 ∙ 52 · 0.27, where $6.13833 represented the price for one tablet),^2^ which was consistent with the fact that multiple entities were offering alendronate at $6 to $9/month.^27^ The brand version of alendronate (ie, Fosamax; Merck, Kenilworth, NJ, USA) was estimated to be $1276 ($38.3525 · 52 · 0.64, where $38.3525 represented the price for one tablet).

We assumed that because of participants' preference or intolerance, not everyone who was initially offered generic oral alendronate would continue the generic version.^10^ In the base case, we assumed that 90% of those who were offered generic alendronate continued to take a generic product, and 10% took the branded product, based on a study using Medicare prescription drug claims data that reported the switching patterns of alendronate (ie, stayed on the branded product, switched to a generic product, and switched to other bisphosphonates) after generic alendronate was released on the market. The study reported that of those who continued bisphosphonates, 10% stayed on the branded product, 89% switched to a generic product, and 1% switched to other bisphosphonates.^28^ This assumption (ie, 10% taking brand product) was supported by a report that brand products (not limited to osteoporosis medications) comprised 10% of all dispensed prescriptions in the US.^29^ We therefore estimated the annual cost of alendronate as $205 ($86 · 0.9 + $1276 · 0.1) in the base case.

There is more uncertainty regarding the cost of teriparatide, so we extensively reviewed the existing literature to estimate the costs of brand and generic/biosimilar teriparatide. The Institute for Clinical and Economic Review (Boston, MA, USA) estimated in their study that the cost of brand teriparatide was 62% (ie, 38% discount) of WAC.^12^ We adopted their assumptions and estimated the annual cost of brand teriparatide as $27,618 ($3426.50 · 13 · 0.62, where $3426.50 represented the WAC for one 600 μg/2.4 mL injection intended for a 28‐day supply). Because generic/biosimilar teriparatide is not yet available in the US, we assumed that the cost of generic/biosimilar teriparatide would be discounted 30% relative to brand [ie, $19,332 ($27,618 · 0.7)], based on a study of generic prices relative to brand prices as a function of time since initial generic entry in the US.^30^ This assumption was supported by an expert opinion of a financial analyst who also estimated that the price of generic teriparatide would be priced at a 30% discount.^31^ Biosimilar prices that were discounted 30% relative to the reference biologic price also appeared to be a reasonable assumption.^32^ We, therefore, estimated the annual cost of teriparatide as $20,161 ($19,332 · 0.9 + $27,618 · 0.1), by applying the same assumption as the cost of alendronate that 10% took the branded product and 90% took a generic/biosimilar product in the base case. It is, however, more challenging to estimate the proportion of brand teriparatide after biosimilar teriparatide becomes available, as currently there is no biosimilar product available to treat osteoporosis in the US market. We assumed that the proportion of brand teriparatide would be as high as 40% (ie, the proportion of biosimilar would be 60%) after biosimilar teriparatide became available^32^ and performed a sensitivity analysis with different proportions of generic/biosimilar products (ie, 90%—base case, 75%, and 60%) to estimate the cost of teriparatide.

The costs of alendronate and teriparatide were proportional to adherence and persistence with treatments. We charged the cost for a 3‐month supply of alendronate or teriparatide (ie, a single prescription filled) for those who discontinued it within the first year.

For the costs of physician visits and DXA scans, we used the allowable charges (Current Procedural Terminology [CPT] codes 99213 and 77080, respectively) based on the national payment amounts from the 2018 Medicare Physician Fee Schedule.^10^, ^33^ We assumed those taking alendronate had a physician visit every year, and those taking teriparatide had a physician visit twice a year. We also included follow‐up DXA scans to be performed at the end of 5 and 10 years for those with alendronate and at the end of 2 years for those with teriparatide.

As future related medical costs, we included the costs of treatments for fractures, such as inpatient, skilled nursing facility, home health, hospice, outpatient, and durable medical equipment, as well as physician/noninstitutional claims.^10^ Future unrelated medical costs were not considered in this analysis because we judged competing risks (for developing conditions other than a fracture) in an osteoporotic woman to be similar between sequential teriparatide/alendronate and alendronate alone.

#### *Informal health care sector*

A patient's time cost was not included, as time costs for daily injection or weekly oral medication were negligible.

#### *Non‐health care sector*

We modeled long‐term care cost associated with hip fractures. We assumed 12% of those who sustained hip fractures remained at a nursing home beyond 1 year and those who remained beyond 1 year required indefinite long‐term care. We conservatively assumed that hip fractures themselves were directly responsible for only 25% of long‐term‐care placements (in other words, long‐term care would have occurred regardless of the hip fracture in 75% of cases).^10^ We used the average cost for a semiprivate room at a nursing home ($6844 /month in 2016, inflated to $7159 in 2018),^34^ and estimated $2577 ($7159 · 12 · 0.12 · 0.25) per year averaged over all participants in the “post‐hip fracture” state until death.^10^ In the year of hip fracture, we modeled 6 months of long‐term‐care cost for those who suffered from hip fracture.

Unpaid lost productivity caused by a hip fracture was included. We used the sex‐, age‐, and race‐specific (65 to 69, 70 to 74, and 75+) rates of labor‐force participation of seniors in 2017 (ie, white women 65 to 69 years old: 27.9%, 70 to 74 years old: 16.6%, 75+ years old: 5.9%),^35^ and median usual weekly earnings of full‐time wages by sex and age in 2018 (women 65+ years old: $757/week).^36^ Bentler and colleagues^37^ reported that after hip fractures 58% were initially discharged to a skilled nursing facility, and Kumar and colleagues^38^ reported that after hip fractures the mean lengths of stay at a hospital and at a skilled nursing facility were 4.9 days and 42.6 days [44.7 days for Medicare fee‐for service (73.7%) and 36.9 days for Medicare Advantage (26.3%)], respectively. We therefore charged 8 weeks of unpaid lost productivity for those who suffered from hip fractures as follows: $1690 ($757 · 8 · 0.279) for ages 65 to 69, $1005 ($757 · 8 · 0.166) for ages 70 to 74, and $357 ($757 · 8 · 0.059) for ages 75 to 80. We did not assume unpaid lost productivity beyond age 80.

### Transition probabilities

#### *Fracture rates*

We modeled incidence rates of hip, clinical vertebral, and wrist fractures based on US hospital discharge data from 2006 and data from Olmsted County, Minnesota, USA, both of which were used in an article that provided updated fracture incidence rates for the US version of the Fracture Risk Assessment Tool (FRAX).^10^ Because the incidence rates of other osteoporotic fractures were not available in this article, we obtained the rates from another published source.^10^ As the target population was those who had prior vertebral fracture, we modeled increased relative risks of second and subsequent vertebral fracture and subsequent hip, wrist, and other fractures associated with prior vertebral fracture.^39^ In addition, we also modeled increased relative risks of fracture for individuals with osteoporosis compared with the general population.^10^ We considered increased risks of fractures associated with both prior vertebral fractures and osteoporosis, as prior history of vertebral fracture and low BMD appear to have a multiplicative effect on fracture risk.^39^

#### *Mortality rates*

We used the 2015 US Vital Statistics Table to obtain annual mortality rates of white women up to age 100,^40^ and we extrapolated these rates up to age 105. We incorporated lifelong excess mortality after a hip fracture, as a meta‐analysis showed that excess mortality appeared to be stable from the second year onward and did not return to the age‐ and sex‐matched baseline even after 10 years. As with long‐term care after a hip fracture, we conservatively assumed that hip fracture events only contribute to 25% of excess mortality, as comorbidities appear to play a large role.^10^ We did not assume excess mortality associated with clinical vertebral fracture, because in our previous study results were very similar to the base case in an alternative scenario where we assumed clinical vertebral fractures had the same impact on extra mortality as hip fractures.^10^

#### *Utilities*

We used EuroQol‐5 Dimension (EQ‐5D) survey results from a sample of the US noninstitutionalized population to obtain age‐ and sex‐specific baseline health state utility values. Fractures are associated with disutility, which is a loss in health‐related quality of life. We assumed that disutilities associated with hip or clinical vertebral fractures were highest in the year immediately following a fracture, but persisted for the rest of life. In contrast, wrist or other osteoporotic fractures incurred a disutility in the first year, but no disutility in subsequent years.^10^ To keep the model parsimonious, we did not consider baseline disutility associated with prior vertebral fracture.

### Model simulation and sensitivity analyses

**Figure 1:**
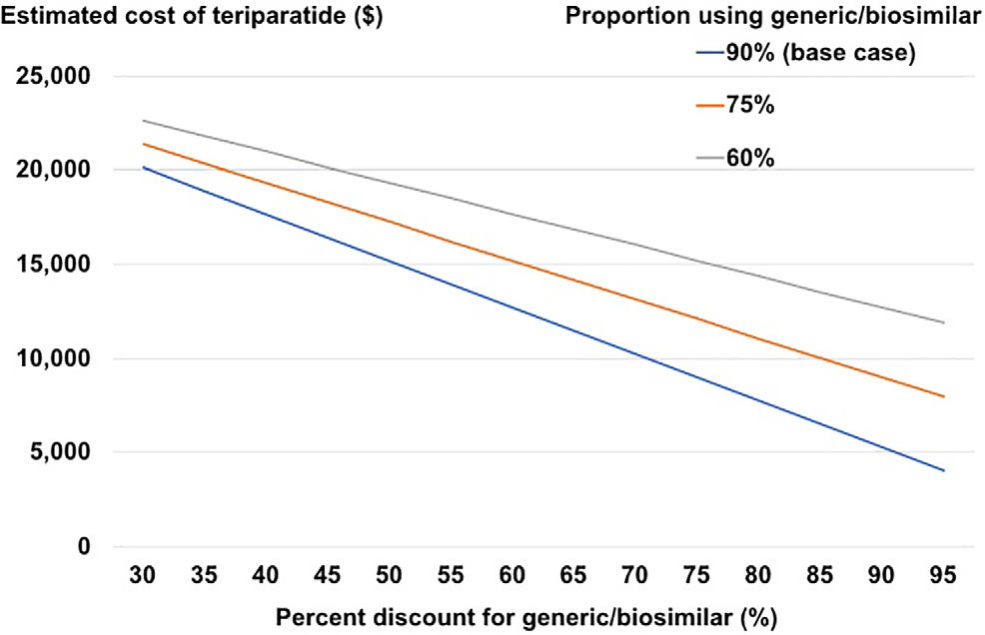
The estimated costs of teriparatide with various assumptions regarding the price discount for generic/biosimilar relative to the brand product and the proportion of individuals using a generic/biosimilar product. The estimated costs of generic/biosimilar teriparatide were decreased in 5% increments compared with the base case assumptions, which was a 30% discount from brand product (ie, the cost was 70% compared with the brand product). The estimated costs of teriparatide were also calculated based on the assumptions that certain proportions (ie, 90%, 75%, or 60%) took a generic/biosimilar product and the rest (ie, 10%, 25%, or 40%) took the brand product.

We performed base case analyses with 100,000 iterations (100,000 individuals through the model one at a time). Next, we conducted deterministic (one‐way) sensitivity analyses in which we evaluated different assumptions for critical model parameters (ie, costs, efficacy, or persistence or adherence of teriparatide) to examine the robustness of the results when the values of the base case assumptions changed. Given the uncertainty in the cost of generic/biosimilar teriparatide at the time of analysis, we performed a special set of deterministic sensitivity analyses that varied the estimated cost of generic/biosimilar teriparatide. Specifically, to determine the threshold costs that made sequential teriparatide/alendronate cost‐effective under the predetermined WTP thresholds (ie, $50,000, $100,000, or $150,000/QALY), we decreased the estimated cost of generic/biosimilar teriparatide in 5% increments compared with the base case assumptions. In addition, we changed the proportions of generic/biosimilar teriparatide to be 60% and 75% (as compared with 90% in the base case) to estimate the annual costs of teriparatide (Fig. 1). Furthermore, we performed a sensitivity analysis, in which we examined the cost‐effectiveness of teriparatide for 2 years followed by alendronate for 5 years compared with alendronate alone for 5 years, otherwise using the same parameters as the base case.

We also performed probabilistic sensitivity analyses, in which parameter values were randomly selected from probability distributions for uncertain key model inputs (Table 2).^10^ To examine how the estimated costs of teriparatide affected cost‐effectiveness, we performed probabilistic sensitivity analyses with different estimated costs of teriparatide as we used in the deterministic sensitivity analyses. Monte Carlo simulations were performed with 1000 simulations and 10,000 trials per simulation.

We performed deterministic and probabilistic sensitivity analyses only from the societal perspective, as the results of the base cases from the societal and health care sector perspectives were very similar, as presented in the Results section. To verify the model's accuracy, we initially included a “no‐intervention” arm to calculate mortality and fracture rates in the model.

## Results

### Model validation

The model predicted that 99.9% died by age 105 regardless of starting age (ie, 65, 70, 75, or 80 years old). Our model also predicted that without an intervention, the probabilities of a woman age 65 with prior vertebral fracture having an additional clinical vertebral fracture or at least one hip, wrist, or other fracture over her lifetime were 58.7%, 59.3%, 25.9%, or 46.6%, respectively. These risks represented a high‐risk population for osteoporotic fracture, as compared with our previous study in the US setting in which our target population was community‐dwelling US white women without a prior major osteoporotic fracture (ie, hip, vertebral, or wrist). In this prior study, the probabilities of a woman age 65 having at least one clinical vertebral, hip, or wrist fracture over her lifetime without an intervention were 11.2%, 19.1%, and 16.0%, respectively.^10^

### Base case analyses

The ICERs of sequential teriparatide/alendronate compared with alendronate alone at ages 65, 70, 75, or 80 were $434,400, $330,000, $280,100, and $290,800 per QALY, respectively, from the societal perspective; and $441,700, $336,700, $288,200, and $299,100 per QALY, respectively, from the health care sector perspective (Table 3).

**Table 3:** The Results of the Base Case Analyses

Perspective	Societal			Health care sector		
	Cost ($)	QALY	ICER ($/QALY)	Cost ($)	QALY	ICER ($/QALY)
Age 65						
Alendronate Alone	42,830	10.55	Reference	33,130	10.55	Reference
Teriparatide/Alendronate	54,060	10.58	434,400	44,560	10.58	441,700
Age 70						
Alendronate Alone	43,390	8.70	Reference	34,200	8.70	Reference
Teriparatide/Alendronate	54,350	8.73	330,000	45,400	8.73	336,700
Age 75						
Alendronate Alone	42,530	7.00	Reference	34,240	7.00	Reference
Teriparatide/Alendronate	53,110	7.04	280,100	45,120	7.04	288,200
Age 80						
Alendronate Alone	39,290	5.31	Reference	32,520	5.31	Reference
Teriparatide/Alendronate	49,580	5.34	290,800	43,120	5.34	299,100

QALY = quality‐adjusted life year, ICER = incremental cost‐effectiveness ratio.

### Deterministic sensitivity analyses

**Figure 2:**
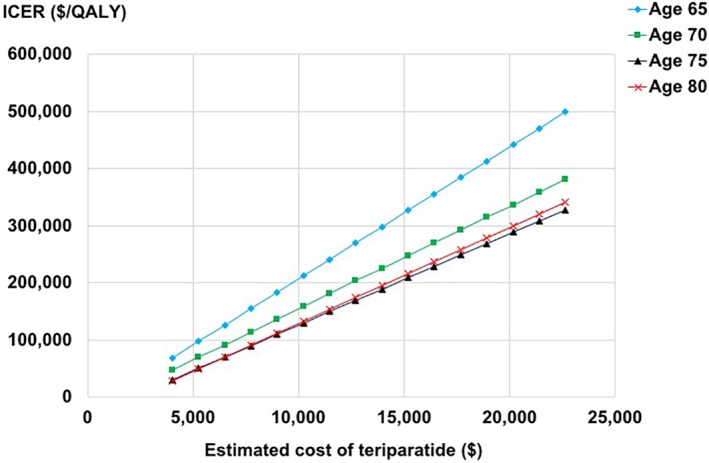
ICER = incremental cost‐effectiveness ratio ($/quality‐adjusted life year). The results of deterministic sensitivity analyses around the costs of teriparatide. The estimated costs of generic/biosimilar teriparatide were decreased in 5% increments compared with the base case assumptions (ie, 70% of brand product). The estimated annual costs of teriparatide were calculated based on the assumption that 90% took generic/biosimilar and 10% took brand products.

In deterministic sensitivity analyses, results were most sensitive to the changes in the estimated cost of teriparatide. For example, if we estimated the cost of generic/biosimilar to be 15% of brand (ie, 85% discount) making the annual cost of teriparatide $6490 for a 65‐year old cohort; or we estimated the cost of generic/biosimilar to be 35% of brand (ie, 65% discount) making the annual cost of teriparatide $11,461 for a 75‐year‐old cohort; the ICERs of sequential teriparatide/alendronate were below the WTP threshold of $150,000/QALY (Fig. 2).

**Figure 3:**
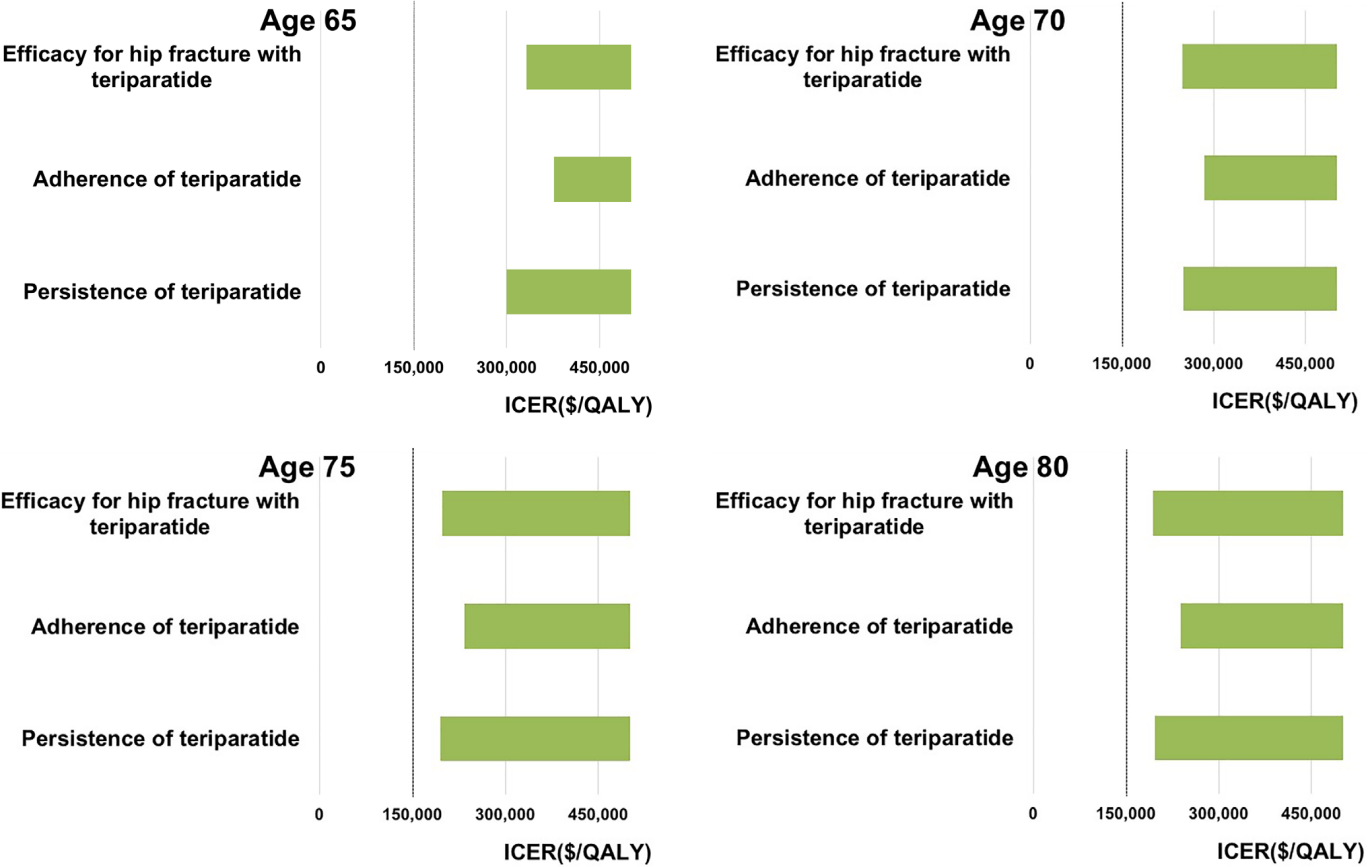
ICER = incremental cost‐effectiveness ratio, QALY = quality‐adjusted life year. The results of deterministic sensitivity analyses for the three most‐influential parameters other than the cost of teriparatide. The figures present the ICERs of sequential teriparatide/alendronate compared with alendronate alone, when the indicated model parameters are varied across their ranges from a societal perspective. The bolded vertical line represents the ICER of $150,000 per QALY. We present the three largest changes of ICERs compared with base case parameter estimates.

Other than changes in costs of teriparatide, we found the efficacy of teriparatide for preventing hip fracture, and the persistence and adherence rates of teriparatide to be the three most influential factors in deterministic sensitivity analyses. However, even if we assumed the relative risk of hip fracture with teriparatide was 0.1 (instead of 0.42 in the base case) or assumed the persistence rate or adherence rate was 1.5 times as high as the base case, ICERs of sequential teriparatide/alendronate were greater than the predetermined threshold of WTP of $150,000/QALY at all ages examined (Fig. 3).

In a sensitivity analysis, in which we examined the cost‐effectiveness of teriparatide for 2 years followed by alendronate for 5 years compared with alendronate alone for 5 years, the results were similar to the base case and the conclusions remained the same.

### Probabilistic sensitivity analyses

**Figure 4:**
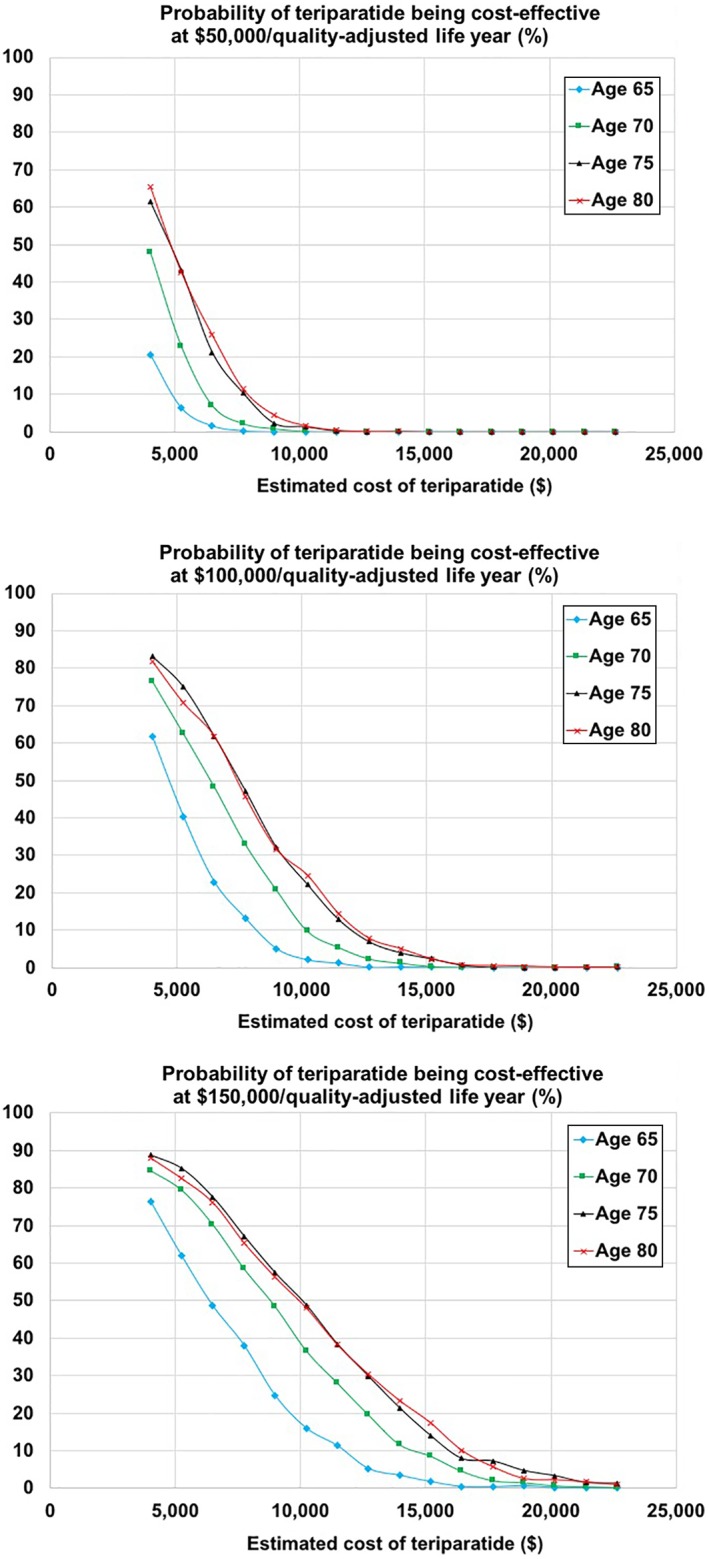
The results of probabilistic sensitivity analyses, stratified by incremental cost‐effectiveness ratio (ICER). The estimated costs of generic/biosimilar teriparatide were decreased in 5% increments compared with the base case assumptions (ie, 70% of brand product). The estimated annual costs of teriparatide were calculated based on the assumption that 90% took a generic/biosimilar product and 10% took brand products.

In probabilistic sensitivity analyses, the probabilities of sequential teriparatide/alendronate being cost‐effective compared with alendronate alone were 0.1%, 0.7%, 3.3%, and 2.2% at ages 65, 70, 75, and 80, respectively, at a WTP of $150,000 per QALY, under the base case assumptions that teriparatide cost was $20,161, in which the cost of a generic/biosimilar product was 70% of the brand product (ie, 30% discount) and 90% took a generic/ biosimilar product. If we assumed that the cost of a generic/biosimilar product was 5% of the branded product (ie, 95% discount) and 90% took a generic/biosimilar product, making the annual cost of teriparatide $4005, the probabilities of sequential teriparatide/alendronate being cost‐effective were 76.4%, 84.7%, 88.7%, and 88.1%, at ages 65, 70, 75, and 80, respectively, at a WTP of $150,000 per QALY (Fig. 4).

## Discussion

To understand the potential health economic impact of the availability of generic/biosimilar teriparatide after August 2019 in the US, we examined the cost‐effectiveness of sequential teriparatide/alendronate compared with alendronate alone among community‐dwelling older osteoporotic women with prior vertebral fractures. In the base case, sequential teriparatide/alendronate was not cost‐effective at all ages examined at a WTP threshold of $150,000/QALY, and the cost of teriparatide was the main factor driving the lack of cost‐effectiveness. Therefore, sequential teriparatide/alendronate would not be cost‐effective unless the cost of a generic/biosimilar product were heavily discounted with respect to the current brand cost.

The key variable in our analyses was the cost of generic/biosimilar teriparatide. In the base case, we estimated the cost of generic/biosimilar teriparatide as 70% of the estimated cost of the brand product (ie, 30% discount), based on Frank and Salkever's study^30^ regarding generic prices relative to brand prices as a function of time since initial generic entry in the US, and also on the expert opinion of a financial analyst.^31^ Similarly, biosimilar prices that were discounted 30% relative to the reference biologic price appeared to be a reasonable assumption.^32^ We then varied the estimated costs of generic/biosimilar teriparatide extensively from 5% to 70% (ie, base case) of brand product to examine the potential influence of costs of teriparatide on cost‐effectiveness, as Frank and Salkever^30^ showed that generic prices declined relative to brand prices over time and became less than 10% of brand (ie, 90% discount). Further, we assumed that 10% took the brand product and the remaining 90% took the generic/biosimilar in the base case. Given the uncertainty surrounding the future market penetration of generic/biosimilar teriparatide in the US, we also explored scenarios in which 75% or 60% took a generic/biosimilar product. By changing the price discount and the proportion of individuals taking generic/biosimilar teriparatide, we varied the annual estimated costs of teriparatide from $4005 to $22,646. In Japan, at 2018 exchange rates, a 28‐day supply of teriparatide for daily use cost $392.40, making the annual cost $5102.^41^, ^42^ Compared with our estimation that brand teriparatide cost of $27,618 in the US, the identical brand teriparatide product in Japan cost only 18.5% of its counterpart in the US. This information shows that teriparatide has the potential to become heavily discounted relative to current brand prices in the US should there be multiple entrants into the generic/biosimilar market.

Our study both confirms and extends prior work in this area. Liu and colleagues' 2006 study^6^ in the US setting compared sequential teriparatide/alendronate (ie, daily s.c. teriparatide for 2 years followed by daily oral alendronate for 5 years in the study) with alendronate alone for 5 years in women with severe osteoporosis, defined as a femoral neck BMD *T*‐score of −2.5 in addition to prior history of vertebral fracture. They showed that sequential teriparatide/alendronate was not cost‐effective, mainly because teriparatide was expensive. As generic versions of alendronate were not available at the time of the study, the cost of alendronate was based on the brand product, making the estimated annual cost of alendronate $894, while that of the brand product of teriparatide was $6720 (costs were presented in 2003 US dollars).^6^ We updated Liu and colleagues' study^6^ in several ways. First, although in their study, both teriparatide and alendronate were available only as a branded product as discussed above, in our study we included a generic version of alendronate and a generic/biosimilar version of teriparatide. In addition, in our model we included persistence and adherence rates for the treatments based on real‐world data, and included more recent estimates of other parameters (ie, efficacy, costs including medications, fracture treatments and others, fracture incidence, utility and disutility, and mortality rates).

Other than Liu and colleagues' study,^6^ there have been three more recent cost‐effectiveness analyses for fracture prevention in the US setting including teriparatide. Unlike our study, however, Hiligsmann and colleagues' and Le and colleagues' studies focused on cost‐effectiveness of sequential treatments of abaloparatide, which was approved in the US in 2017, followed by alendronate, compared with teriparatide followed by alendronate.^13^, ^14^ The Institute for Clinical and Economic Review's study examined the cost‐effectiveness of teriparatide compared with zoledronate or no treatment.^12^ In this study, the ICER of teriparatide compared with no treatment would be less than $150,000/QALY if the annual cost of teriparatide were $5474 in 2016 dollars, an approximately 77% discount from their cost in base case. Their results were consistent with our finding that the cost of teriparatide would need to be heavily discounted with respect to the current brand cost for sequential teriparatide/alendronate to be cost‐effective compared with alendronate alone. None of these studies focused on the potential cost‐effectiveness of sequential teriparatide/alendronate compared with alendronate after generic/biosimilar teriparatide availability in the US as we did.

In our previous study, in which we examined cost‐effectiveness of s.c. denosumab every 6 months for 5 years compared with oral alendronate weekly for 5 years for osteoporotic older women in Japan, we concluded denosumab was cost‐effective or even cost‐saving mainly based on denosumab's higher persistence leading to higher efficacy.^11^ In this analysis, however, even when we assumed a higher efficacy of teriparatide or higher persistence or adherence rates of teriparatide in the deterministic sensitivity analyses, sequential teriparatide/alendronate did not become cost‐effective at any of the predetermined WTP thresholds. As ICERs were determined by the ratios of cost and effectiveness, teriparatide was estimated to be so expensive even after the potential introduction of generic/biosimilar versions that higher efficacy or higher persistence or adherence rates of teriparatide than we assumed in the base case were not enough to compensate for the high cost of teriparatide.

We note several limitations. First, although persistence and adherence rates are known to be important parameters in cost‐effectiveness analyses of osteoporotic fracture prevention, we estimated these values based on a single observational study in the US. However, we performed deterministic sensitivity analyses that assumed the persistence or adherence rates of teriparatide could be 1.5 times higher or lower than those in the base case. Second, to keep the model parsimonious, we did not simulate some contraindications (eg, avoidance of alendronate for those with a creatinine clearance <30 mL/min) or adverse events (eg, hypercalcemia with teriparatide).^1^ However, serious adverse events caused by teriparatide are considered to be rare; therefore, they were unlikely to impact the results of our cost‐effectiveness analyses.^12^ Third, alendronate was prescribed after completion of teriparatide in our analysis. However, another medication such as denosumab can be prescribed afterward instead of alendronate,^43^ which was beyond the scope of our analysis. Finally, in terms of the generalizability of this study, it is important to note that our analysis was based on the current cost of brand teriparatide in the US. Therefore, the results of our study may not be applicable to other countries. In addition, it is important to interpret the results of our cost‐effectiveness analysis as focusing on a high‐risk population in the US (ie, osteoporotic white US women with a prior history of vertebral fracture).

Despite these limitations, our study has notable strengths. We identified that the main driver of sequential teriparatide/alendronate not being cost‐effective was the cost of teriparatide; therefore, we worked meticulously to estimate the costs of teriparatide accurately. We then conducted extensive analyses of potential cost‐effectiveness including various price discounts for generic/biosimilar teriparatide compared with the cost of the brand product, and also with different rates of market penetration for generic/biosimilar teriparatide. In addition, we were able to conduct this analysis without external funding, which might have generated conflicts of interest.

In conclusion, among community‐dwelling older osteoporotic women with prior vertebral fracture, sequential teriparatide/alendronate is likely not cost‐effective compared with alendronate alone even with the potential availability of generic/biosimilar teriparatide in the US market under the assumption that the cost of generic/biosimilar teriparatide would be 70% (ie, 30% discount) of the brand product and 90% took the generic/ biosimilar product. Sequential teriparatide/alendronate would not be cost‐effective unless the cost of teriparatide were heavily discounted with respect to the current brand cost.

## Disclosures

Takahiro Mori: The joint appointment as an associate professor at the University of Tsukuba was sponsored by JMDC Inc. in the 2018 financial year (ie, April 2018 to March 2019), and by SMS CO., LTD. in the 2019 financial year (ie, April 2019 to the present). Neither JMDC Inc. nor SMS CO., LTD played any role in the conduct of this study. Carolyn J Crandall and David A Ganz declare that they have no conflict of interest.

## Supporting information


**Supplemental Table S1.** Osteoporosis‐Specific Checklist—Specific Items to Include When Reporting Economic Evaluations on Osteoporosis.
