# Supplementary material for: Cost‐Effectiveness of Sequential Teriparatide/Alendronate Versus Alendronate‐Alone Strategies in High‐Risk Osteoporotic Women in the US: Analyzing the Impact of Generic/Biosimilar Teriparatide
Source: JBMR Plus. 2019 Nov 13;3(11):e10233. doi: 10.1002/jbm4.10233 (PMC6874180; doi:10.1002/jbm4.10233)
Supplement: Supplementary file 1 — Supplemental Table S1. Osteoporosis‐Specific Checklist—Specific Items to Include When Reporting Economic Evaluations on Osteoporosis. [file JBM4-3-na-s001.docx]

**Supplemental Table 1**

Osteoporosis-specific checklist—specific items to include when reporting economic evaluations on osteoporosis

| **Item** | **Item no.** | **Recommendation** | **Reported on page no./line no.** |
| --- | --- | --- | --- |
| Transition probabilities | 1 | Report the transition probabilities and how they were estimated (including increased fracture risk) | Method section |
| Excess mortality after fractures | 2 | Describe approaches and data sources used for the excess mortality after fractures | Method section |
| Fractures costs | 3 | Describe approaches and data sources used for fractures costs | Method section |
| Fractures effects on utility | 4 | Describe approaches and data sources used for the effects of fractures on utility | Method section |
| Treatment effect during treatment | 5 | Describe fully the methods used for the identification, selection, and synthesis of clinical effectiveness data (per fracture site) | Method section |
| Treatment effect after discontinuation | 6 | Describe fully the methods used for the treatment effect after discontinuation | Method section |
| Medication adherence | 7 | Describe approaches and data sources used for modeling medication adherence | Method section |
| Treatment costs | 8 | Describe approaches and data sources used for therapy costs | Method section |
| Treatment side effects | 9 | Describe approaches and data sources used for costs and utilities effects of adverse events | Discussion section |
